# Clinical features, diagnosis, and treatment of primary Sjögren’s disease with interstitial lung disease: A narrative review

**DOI:** 10.3389/fimmu.2026.1806321

**Published:** 2026-04-14

**Authors:** Runzhi Liu, Linlin Zhi, Yuanfang Liang, He Jiang, Wenqing Xin, Ni Yan, Xiaoyan Li

**Affiliations:** 1 Department of Rheumatology and Immunology, Shaanxi Provincial People’s Hospital, Xi’an, Shaanxi, China; 2 Department of Rheumatology and Immunology, Xi’an Medical University, Xi’an, Shaanxi, China

**Keywords:** anti-Ro52 antibody, biomarkers, interstitial lung disease, nintedanib, prognosis, rituximab, Sjögren’s disease

## Abstract

Interstitial lung disease (ILD) is a severe and frequent extraglandular manifestation of primary Sjögren’s disease (pSjD), conferring significant morbidity and mortality. This narrative review synthesizes current evidence on the epidemiology, pathogenesis, clinical phenotypes, diagnosis, and management of pSjD-ILD, with a focus on phenotype-stratified care and evidence limitations. Key risk factors include anti-Ro52 antibody seropositivity, advanced age, and male sex. Diagnosis relies on a multidisciplinary approach integrating clinical assessment, serology, high-resolution computed tomography (predominantly fibrotic nonspecific interstitial pneumonia pattern), and pulmonary function tests. Pathogenesis involves a complex interplay of autoantibody-mediated damage, immune cell dysregulation, and dysbalanced pro-fibrotic signaling. We emphasize a phenotype-stratified treatment strategy: immunosuppression forms the cornerstone for inflammatory-predominant disease, while antifibrotic agents are pivotal for progressive fibrotic phenotypes. Critical limitations of current evidence include the extrapolation of most therapeutic data from other connective tissue disease-associated ILDs (CTD-ILDs) and a lack of pSjD-ILD-specific randomized controlled trials (RCTs). Emerging therapies, including rituximab and nintedanib, show promise but require further validation in pSjD-ILD cohorts. This review provides a pragmatic clinical framework to guide diagnosis, risk stratification, and individualized management, while highlighting critical unmet needs for future research, such as validated prognostic scores and pSjD-ILD-specific clinical trials.

## 1 Introduction

Primary Sjögren’s disease (pSjD) is a chronic autoimmune disorder characterized by lymphocytic infiltration of exocrine glands, leading to classic sicca symptoms (1–12). Extraglandular manifestations are common, with interstitial lung disease (ILD) representing the most significant pulmonary complication (13–18). ILD affects approximately 13–20% of pSjD patients, doubles mortality risk, and frequently presents as the initial manifestation of occult pSjD—in up to 83.3% of cases—challenging traditional diagnostic paradigms (1, 19–21).

This narrative review synthesizes current knowledge on pSjD-ILD, encompassing epidemiology, pathogenesis, clinical features, diagnosis, and management. Based on a systematic literature search of peer-reviewed articles and guidelines focused on pSjD-ILD, we prioritize evidence from original research and meta-analyses; data from other connective tissue disease-associated ILDs are included only for therapeutic extrapolation, with explicit acknowledgment of their limitations.

Despite its clinical importance, pSjD-ILD research is constrained by heterogeneous study designs, absence of standardized diagnostic criteria, and a lack of disease-specific randomized controlled trials (22). Most therapeutic and prognostic data are extrapolated from systemic sclerosis-associated ILD or idiopathic pulmonary fibrosis, and no validated pSjD-ILD-specific prognostic score exists (16). This review addresses these gaps by proposing a phenotype-stratified clinical framework, clarifying biomarker utility, and explicitly highlighting evidence limitations to inform clinical practice and guide future research.

### 1.1 Epidemiological characteristics

Epidemiological data for pSjD-ILD are heterogeneous due to differing study designs, criteria, and populations, yet consistent patterns in prevalence and risk factors are recognized.

ILD is the most frequent serious pulmonary complication in pSjD, with an overall estimated prevalence of ~20% in international cohorts and 13% in a Chinese single-center study (1, 13, 23–25). Of these patients, 11–33% require long-term oxygen therapy, reflecting significant functional impairment (20). Contrary to the traditional view of ILD as a late pSjD manifestation, contemporary evidence shows heterogeneous onset: ILD symptoms precede or coincide with pSjD diagnosis in 10–51% and up to 83.3% of cases, respectively. This highlights ILD as a potential initial presentation of occult pSjD and necessitates clinical suspicion for underlying Sjögren’s disease in patients with ILD of unknown cause (13, 26).

Age and sex are established demographic risk factors. The average age at pSjD-ILD diagnosis is ~69.5 years, with significantly higher prevalence in elderly-onset pSjD (26, 27). Male sex is an independent risk factor for mortality, with cohort studies showing significantly lower survival in male patients. Furthermore, postmenopausal females are at elevated risk, potentially due to hormonal influences on immune homeostasis (28).

In addition to these demographic factors, anti-Ro52 seropositivity represents the strongest serological risk factor for pSjD-ILD. It is present in 51.2–90% of affected patients and is independently associated with increased fibrosis severity and reduced overall survival (15, 29, 30). This serological marker not only identifies patients at higher risk of developing ILD but also stratifies those with more aggressive disease trajectories, making it a key tool for clinical risk assessment.

## 2 Pathogenesis

The pathogenesis of pSjD-ILD involves a complex interplay of autoimmune dysregulation, inflammatory injury, and progressive fibrosis. These links interact to form a complex pathological network.

### 2.1 Autoantibody-mediated immune damage

Autoantibodies, particularly anti-Ro52, are central to the pathogenesis of pSjD-ILD. Present in 70–90% of pSjD patients, anti-Ro52 positivity—especially when isolated or combined with anti-Ro60—is strongly associated with ILD risk and independently predicts reduced survival (15, 30–32).

Anti-Ro52 antibodies exert profibrotic effects by targeting pulmonary epithelial cells and fibroblasts. Their binding to epithelial cells activates the NLRP3 inflammasome, leading to IL-1β release (33–36). IL-1β then induces HLA-DR upregulation on interstitial lung antigen-presenting cells, which in turn activate Ro52-specific CD4+ Th1 and CD8+ cytotoxic T lymphocytes (37). These Th1 cells amplify inflammation and fibrosis by secreting IFN-γ, TNF-α, and IL-12, and by directly engaging fibroblasts via CD40, thereby activating the NF-κB pathway and driving fibrotic progression (38, 39).

**Figure 1:**
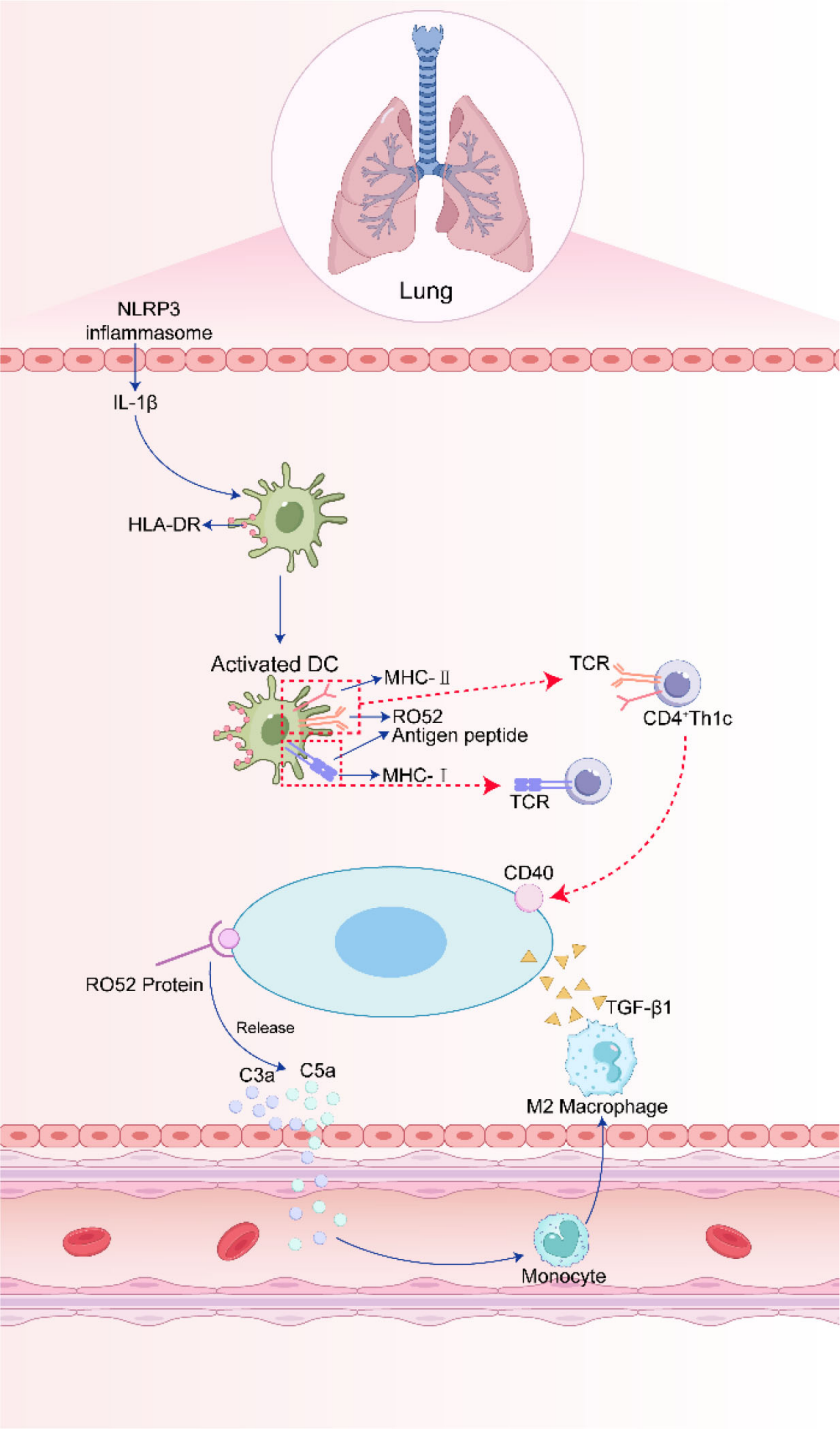
Schematic of anti-Ro52 antibody-mediated pathogenic mechanisms in pSjD-ILD. Anti-Ro52 antibodies bind to pulmonary epithelial cells, activating the NLRP3 inflammasome and releasing IL-1β, which promotes activation of Ro52-specific Th1/CD8+ T cells. Th1 cells secrete pro-inflammatory cytokines (IFN-γ, TNF-α) and activate fibroblasts via CD40-NF-κB signaling. Additionally, anti-Ro52 immune complexes on fibroblasts trigger the classical complement pathway, recruiting monocytes that differentiate into M2 macrophages (secreting TGF-β1) to drive fibroblast activation and collagen synthesis via SMAD3/AKT/mTOR pathways. AKT, protein kinase B; HLA-DR, human leukocyte antigen – DR isotype; IFN-γ, interferon-γ; IL, interleukin; NF-κB, nuclear factor kappa B; NLRP3, NACHT, LRR, and PYD domains-containing protein 3; mTOR, mammalian target of rapamycin; SMAD3, SMAD family member 3; TGF-β1, transforming growth factor-β1; Th1, T helper 1; TNF-α, tumor necrosis factor-α.

Concurrently, anti-Ro52 antibodies form immune complexes on lung fibroblasts, activating the classical complement pathway and generating C3a and C5a, which recruit monocytes. These monocytes differentiate into M2 macrophages that secrete TGF-β1. TGF-β1 subsequently induces SMAD3 phosphorylation and activates the AKT/mTOR pathway in fibroblasts, promoting type I collagen synthesis and fibrosis (30, 40). Furthermore, anti-Ro52 positivity correlates with increased MBD2 expression in lung fibroblasts, likely through NF-κB activation, potentially amplifying profibrotic signaling via the TGF-β/Smad and PI3K/AKT/mTOR pathways (**Figure 1**) (41–43).

Other autoantibodies also contribute synergistically. Anti-SSB/La titers correlate with reduced DLCO, and its presence—particularly within a quadruple-positive serology (anti-SSA/Ro60, anti-Ro52, anti-SSB/La)—increases the risk of lymphocytic interstitial pneumonia (44, 45). Additionally, antibodies against U1-70K (~30%) and KS (~20%) in pSjD-ILD may exacerbate local immune responses through epitope spreading against pulmonary ribonucleoproteins (46, 47). The detailed pathogenic cascade of anti-Ro52 antibodies in pSjD-ILD is illustrated in **Figure 1**.

### 2.2 Immune cell dysfunction

Immune cell dysregulation is central to pSjD-ILD pathogenesis, involving intricate interactions among T cells, B cells, and stromal cells.

In pSjD-ILD, CD8+ T cells in bronchoalveolar lavage fluid exhibit an exhausted phenotype with high CD69 and PD-1 expression, while CD4+ T cells show aberrant memory differentiation and impaired mitochondrial metabolic adaptation (31, 37). A pronounced Th1/Th2 imbalance is observed, characterized by increased Th1 differentiation and reduced circulating Th2 proportion. This shift drives inflammation and fibrosis through dual mechanisms: Th1-derived IFN-γ activates the fibroblast JAK-STAT pathway, upregulating collagen synthesis; concurrently, diminished Th2-derived IL-4 secretion weakens a key inhibitory signal on Th1 responses, further amplifying the Th1-dominant state (31, 48–50).

B cells contribute critically through local autoantibody production and cytokine-mediated crosstalk. Clonally expanded B cells within the lung interstitium secrete autoantibodies (e.g., anti-Ro52, anti-RNP), with higher titers in BALF than serum, indicating local production (46). These B cells also secrete BAFF, sustaining autoreactive B-cell survival and promoting Th17 differentiation (46). Th17-derived IL-17A upregulates fibroblast CXCL10, establishing a feed-forward loop: CXCL10 recruits CXCR3+ Th1 cells, whose IFN-γ further stimulates CXCL10 production, amplifying Th1 recruitment. IFN-γ also acts on macrophages via CXCR3 to release TNF-α and IL-6, perpetuating chronic inflammation. Additionally, B-cell subsets secrete IL-6, promoting fibroblast proliferation via STAT3 (51, 52).

These interconnected mechanisms provide the rationale for B-cell depletion therapy. The anti-CD20 antibody rituximab improves lung function in pSjD-ILD not only by reducing pathogenic autoantibodies but also by disrupting the B-cell-dependent inflammatory and fibrotic loops that sustain disease progression.

### 2.3 Dysregulated fibrotic signaling and epigenetic mechanisms

Pulmonary fibrosis, the key pathology underlying end-stage respiratory failure in pSjD-ILD, arises from an imbalance that favors pro-fibrotic over anti-fibrotic pathways, coupled with aberrant fibroblast behavior.

**Figure 2:**
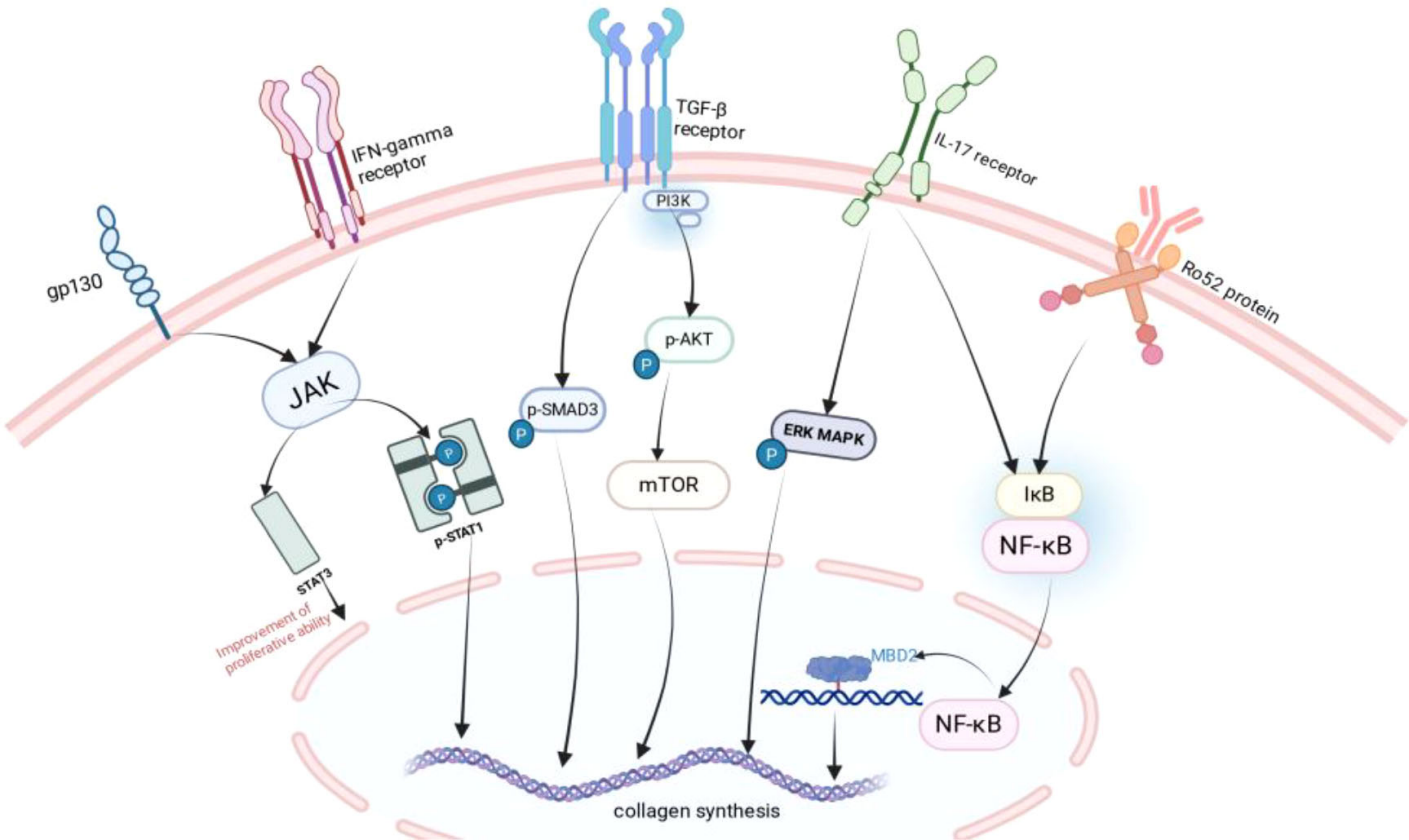
Key fibrotic signaling pathways and epigenetic regulation in pSjD-ILD fibroblasts. Fibroblasts activate multiple synergistic signaling axes via membrane receptors (TGF-βR, IFN-γR, IL-17R, gp130) and Ro52 protein: TGF-βR mediates SMAD3 phosphorylation (canonical) and PI3K-AKT-mTOR activation (non-canonical); IL-17R/IFN-γR drives ERK MAPK/STAT activation; Ro52 promotes NF-κB-MBD2 complex formation. These pathways collectively upregulate collagen synthesis. The AHR-PTEN antifibrotic axis is silenced by MBD2-mediated AHR promoter methylation. AKT, protein kinase B; ERK, extracellular signal-regulated kinase; IFN-γ, interferon-γ; IκB, inhibitor of κB; IL-17, interleukin-17; IL-17R, interleukin-17 receptor; JAK, Janus kinase; MAPK, mitogen-activated protein kinase; MBD2, methyl-CpG-binding domain protein 2; mTOR, mammalian target of rapamycin; NF-κB, nuclear factor kappa B; PI3K, phosphoinositide 3-kinase; STAT, signal transducer and activator of transcription; TGF-β, transforming growth factor-β; TβR, transforming growth factor-β receptor.

The master pro-fibrotic regulator, TGF-β, drives collagen deposition primarily by activating the AKT/mTOR pathway in fibroblasts (**Figure 2**) (53). Conversely, the tryptophan metabolite kynurenine exerts anti-fibrotic effects by entering fibroblasts and binding to the aryl hydrocarbon receptor (AHR). Upon nuclear translocation, AHR upregulates PTEN, which inhibits AKT phosphorylation, thereby suppressing fibroblast activation and the synthesis of α-SMA and collagen (53). This AHR-PTEN axis thus serves as a critical checkpoint against fibrosis. In pSjD-ILD, however, this protective axis is epigenetically silenced. Upregulated MBD2 methylates the AHR promoter, rendering fibroblasts insensitive to kynurenine. Disabling this anti-fibrotic checkpoint permits unchecked TGF-β-driven fibrosis (53).

Adding to the complexity, single-cell studies have identified expanded pathogenic fibroblast subsets in pSjD-ILD lungs. These include CXCL10^+^CCL19^+^ immune-interacting fibroblasts that recruit inflammatory cells, and SPARC^+^COL3A1^+^ vascular-interacting fibroblasts that remodel the extracellular matrix, collectively driving both inflammation and fibrosis (54).

Further amplifying the pro-fibrotic milieu, elevated serum TGF-β1 correlates with increased Angptl2. Angptl2 may promote perivascular fibrosis and endothelial dysfunction, contributing to impaired DLCO, and enhance fibroblast contractility via the Rho/ROCK pathway, potentially influencing FVC reduction (55). Together, these dysregulated signaling pathways and epigenetic modifications highlight potential therapeutic targets, including the MBD2-AHR-PTEN axis and specific fibroblast subsets. The integrated regulatory network of fibrotic signaling and epigenetic modification is summarized in **Figure 2**.

### 2.4 Gut–lung axis dysbiosis

Emerging evidence implicates gut microbiota dysbiosis in the pathogenesis of pSjD-ILD through two interconnected mechanisms: metabolic interference and molecular mimicry.

Patients with pSjD-ILD exhibit an altered gut microbiota characterized by enrichment of the L-phenylalanine biosynthesis pathway, increased abundance of *Lactobacillus salivarius*, and decreased *Clostridium ruminantium*. *L. salivarius* metabolizes L-phenylalanine to phenylacetic acid (PAA), which, upon systemic absorption, engages the aryl hydrocarbon receptor (AHR) on lung dendritic cells. This engagement shifts the IL-23/IL-10 balance toward pro-inflammatory Th17 differentiation. Th17-derived IL-17A subsequently stimulates fibroblast CCL2 expression, recruiting monocytes that differentiate into pro-fibrotic M2 macrophages, thereby directly linking gut-derived metabolites to pulmonary inflammation and fibrosis (56).

In parallel, the gut microbiota of pSjD patients carries an increased repertoire of genes encoding peritrichous flagella and fimbriae. Peptides derived from these bacterial structures can mimic pSjD autoantigens, priming autoreactive B cells within the gut mucosa. These primed B cells then migrate to the lung, where they undergo clonal expansion and produce pathogenic autoantibodies such as anti-Ro52, thereby initiating or amplifying local autoimmune responses.

Together, these gut–lung axis mechanisms highlight how intestinal dysbiosis may contribute to both the induction and perpetuation of pulmonary autoimmunity and fibrosis in pSjD-ILD.

## 3 Clinical features

The clinical phenotype of pSjD-ILD is heterogeneous, with symptoms, imaging, and laboratory findings correlating with disease stage and fibrosis severity. Sicca symptoms are often subtle (especially when ILD is the initial presentation), with respiratory complaints dominating clinical care (57).

### 3.1 Clinical manifestations

Clinical presentation reflects both pSjD and pulmonary involvement. While hallmark sicca symptoms may be subtle, especially when ILD presents first, respiratory complaints often dominate over mild glandular involvement (58).

Pulmonary symptoms primarily include a dry or minimally productive cough and progressive exertional dyspnea, sometimes accompanied by chest pain and fatigue. Severe cases may present with hypoxemia at rest (57, 59). Approximately 21.5% of pSjD patients report chronic respiratory symptoms, with around 11.9% of them being diagnosed with ILD (57). Persistent respiratory symptoms in pSjD patients warrant investigation for underlying ILD.

Raynaud’s phenomenon is more common in symptomatic pSjD and shows a positive correlation with ILD risk, providing a useful clinical clue for screening (28, 57).

### 3.2 Imaging features

HRCT is the cornerstone imaging modality for pSjD-ILD, revealing patterns that mirror pathological subtypes. The most frequent pattern is fibrotic NSIP (29–52.9%), followed by UIP (17.6–43%), OP (11.8–23.5%), and LIP (5.9–10%) (13, 20, 26, 60, 61). Characteristic HRCT findings include bilateral, symmetric ground-glass opacities and fine reticulation with a mid-to-lower lung predominance in NSIP; subpleural reticulation, honeycombing, and traction bronchiectasis in UIP; and numerous thin-walled cysts, centrilobular nodules, and ground-glass opacities, often with mediastinal lymphadenopathy, in LIP (26, 62).

Longitudinal HRCT shows disease progression in approximately 48.7% of patients, typically characterized by worsening reticulation, progressive traction bronchiectasis, and regression of ground-glass opacities (62). The UIP pattern is critically an independent risk factor for disease progression and poorer prognosis. Complementing HRCT, lung ultrasound (LUS) serves as a valuable, non-invasive screening tool, detecting key findings such as increased B-lines and pleural irregularity (63). A posterior-inferior pleural irregularity score demonstrates diagnostic accuracy (sensitivity 86.6%, specificity 84.2%) and correlates with both the HRCT Warrick score and PFT parameters (64, 65). Quantitative lung analysis (QLA) based on CT densitometry—assessing mean lung density and voxel indices in the -200 to -700 HU range—effectively distinguishes limited from extensive disease (66, 67).

### 3.3 Pulmonary function and functional assessment

Pulmonary function tests (PFTs) constitute the cornerstone of physiological assessment in pSjD-ILD, typically revealing a restrictive ventilatory defect with impaired gas exchange (62). Patients with extensive disease exhibit significantly lower forced vital capacity (FVC) and diffusing capacity for carbon monoxide (DLCO) compared to those with limited involvement (62). Notably, DLCO decline often precedes FVC reduction, serving as an early marker of lung involvement and correlating with emphysematous changes (62). Both reduced FVC and DLCO are independent risk factors for increased mortality (68–70).

The differential response to treatment provides important clinical insights: following immunosuppressive therapy, FVC may stabilize or show modest improvement, whereas DLCO typically recovers slowly. This dissociation suggests that underlying fibrotic progression may persist despite effective control of inflammation (62).

The six-minute walk test (6MWT) offers complementary functional assessment by quantifying exercise tolerance and detecting occult exercise-induced hypoxemia (SpO_2_ < 88%) (71, 72). It also serves as a valuable tool for monitoring treatment response: an increase of ≥ 50 meters in six-minute walk distance (6MWD) is considered a clinically meaningful improvement (73).

Echocardiography is essential for screening pulmonary hypertension (PH)—a devastating complication of pSjD-ILD associated with poor prognosis (74). Key parameters include tricuspid annular plane systolic excursion (TAPSE) and estimated pulmonary artery systolic pressure (sPAP). TAPSE < 17 mm or sPAP > 35 mmHg identifies patients with asymptomatic PH and predicts increased mortality, enabling early intervention (74, 75).

Together, these assessments—PFTs, 6MWT, and echocardiography—provide a comprehensive, multidimensional evaluation of disease severity, treatment response, and complications, forming the foundation for personalized management in pSjD-ILD.

### 3.4 Biomarkers

Laboratory findings in pSjD-ILD encompass autoantibodies, inflammatory markers, and stratified biomarkers that aid diagnosis, risk stratification, and treatment response assessment. These can be categorized into two core types: biomarkers for prognostic stratification and dynamic monitoring, and auxiliary inflammatory risk markers for supplementary assessment. **Table 1** summarizes key biomarkers and their clinical utility.

**Table 1:** Stratified biomarkers in pSjD-ILD (prognostic, monitoring and inflammatory/general risk markers).

Biomarker classification	Specific biomarker	Key clinical utility & cutoff (if validated)	Correlation with pSjD-ILD
Prognostic Biomarkers (predict disease progression/mortality)	Anti-Ro52 Antibody	Seropositivity (51.2–90% prevalence in pSjD-ILD)	Independent predictor of reduced survival, increased fibrosis severity; identifies high-risk disease trajectory
Prognostic Biomarkers	KL-6	>1000 U/mL (100% specificity for pSjD-ILD mortality)	Reflects fibroblast activation; correlates with advanced lung fibrosis
Prognostic Biomarkers	Angptl2	No validated cutoff	Inversely correlates with FVC/DLCO; positively correlates with anti-Ro52/TGF-β1; reflects fibrotic extent
Prognostic Biomarkers	CA50	No validated cutoff	Independent predictor of disease progression and fibrotic worsening
Prognostic Biomarkers	CEA	No validated cutoff	Correlates with HRCT fibrosis severity and progressive fibrosis
Monitoring Biomarkers (track disease activity/treatment response)	CA 19-9	No validated cutoff	Inversely correlates with FVC; declines with effective anti-inflammatory/antifibrotic therapy
Monitoring Biomarkers	CA 125	No validated cutoff	Correlates with HRCT disease severity; normalizes with disease stabilization
Monitoring Biomarkers	MUC5AC	No validated cutoff	Reflects CTD-ILD severity; declines with successful treatment
Monitoring Biomarkers	CRP/CRP-to-Lymphocyte Ratio (CLR)	No validated cutoff	Markers of systemic inflammation; correlate with Th2 cell activity and inflammatory flares; track treatment response for inflammatory-predominant disease
Inflammatory/General Risk Markers (auxiliary risk stratification)	Anti-SSB/La Antibody	10.0–30% prevalence in pSjD-ILD	Correlates with reduced DLCO; no independent prognostic value
Inflammatory/General Risk Markers	Hypoalbuminemia	No validated cutoff	Independent poor prognostic factor; reflects malnutrition and chronic disease burden

CTD-ILD, connective tissue disease-associated interstitial lung disease; CEA, carcinoembryonic antigen; CRP, C-reactive protein; DLCO, diffusing capacity for carbon monoxide; FVC, forced vital capacity; HRCT, high-resolution computed tomography; pSjD-ILD, primary Sjögren’s disease-associated interstitial lung disease; TGF-β1, transforming growth factor-β1.

All biomarkers require correlation with clinical, radiological (HRCT) and physiological (PFTs) findings for comprehensive assessment; validated cutoffs are lacking for most monitoring markers and require further prospective validation in pSjD-ILD-specific cohorts.

#### 3.4.1 Prognostic and monitoring biomarkers

Prognostic biomarkers with validated cutoffs in pSjD-ILD cohorts include anti-Ro52, which has a prevalence of 51.2–90% and serves as an independent predictor of reduced survival and fibrosis severity (15, 29, 30). KL-6 exceeding 1000 U/mL demonstrates 100% specificity for pSjD-ILD mortality and reflects fibroblast activation (68, 76, 77). Angptl2 inversely correlates with FVC and DLCO while positively correlating with anti-Ro52 and TGF-β1, reflecting fibrotic extent (55). Among tumor markers, elevated serum CA50 is an independent predictor of disease progression in pSS-ILD, while increased CEA levels correlate with HRCT fibrosis severity and fibrotic progression (78).

Monitoring biomarkers track dynamic changes in disease activity and treatment response, with serial measurement recommended for high-risk patients. CA 19–9 inversely correlates with FVC and declines with effective anti-inflammatory or anti-fibrotic therapy. CA 125 correlates with HRCT severity and normalizes with disease stabilization (78). MUC5AC reflects CTD-ILD severity and declines with successful treatment (79). Additionally, CRP and the CRP-to-lymphocyte ratio (CLR) serve as markers of systemic inflammation, correlating with Th2 cell activity and inflammatory flares (31, 80).

#### 3.4.2 Auxiliary inflammatory risk markers

Elevated CRP and CLR indicate active systemic inflammation (31, 80), while hypoalbuminemia and anemia are independent poor prognostic factors reflecting malnutrition and chronic disease burden (31, 80). Anti-SSB/La, with a prevalence of 10.0–30%, correlates with reduced DLCO but lacks independent prognostic value (44), serving as a supplementary marker for risk stratification only.

## 4 Diagnostic methods

Diagnosis of pSjD-ILD adheres to a multidisciplinary team (MDT) core principle, with joint consensus by rheumatologists, pulmonologists, and radiologists as the gold standard for definitive diagnosis. Given that ILD often precedes or coincides with classic sicca symptoms of pSjD, the diagnostic process prioritizes the integration of clinical phenotypic features, serological markers, imaging findings and physiological assessments, avoiding misdiagnosis of occult pSjD in patients with isolated ILD manifestations. This section details the stepwise diagnostic approach for pSjD-ILD, including initial clinical and serological screening, key imaging and physiological evaluation, and pathological confirmation for atypical cases.

### 4.1 Initial clinical and serological assessment

Initial clinical assessment focuses on sicca symptoms (xerostomia, xerophthalmia), respiratory complaints (dry cough, dyspnea), and extraglandular manifestations (e.g., Raynaud’s phenomenon). The temporal relationship is key, as ILD often precedes or coincides with sicca symptoms (1, 81).

Initial laboratory screening should include classic pSjD antibodies (anti-SSA/Ro60, anti-SSB/La), with anti-Ro52 being particularly valuable for ILD risk stratification. Also relevant are anti-U1-70K (~30%) and anti-KS (~20%) (47, 68, 77, 82).

### 4.2 Imaging and physiological evaluation

HRCT is central to diagnosing ILD, with key patterns including fibrotic NSIP, UIP, OP, and LIP. Features such as mediastinal lymphadenopathy or thin-walled cysts can distinguish pSjD-ILD from IPF (13, 20, 59, 83). Lung ultrasound serves as a non-radiation screening tool; a posterior-inferior pleural irregularity index ≥15 points suggests ILD. Pulmonary function tests (FVC and DLCO) are essential for functional assessment (64, 65). FVC <80% or DLCO <70% predicted should prompt HRCT evaluation. Guidelines recommend serial PFTs every 6–12 months in pSjD, with abnormal results warranting HRCT (70, 84).

### 4.3 Pathological confirmation and MDT integration

Histopathology supports diagnosis. A minor salivary gland biopsy with a focal lymphocytic sialadenitis focus score ≥1 is valuable, particularly in seronegative or paucisymptomatic patients with ILD (1, 85, 86). Surgical lung biopsy is reserved for atypical cases to differentiate from IPF or malignancy (87, 88).

Final diagnosis requires MDT consensus among rheumatologists, pulmonologists, and radiologists. For patients with interstitial pneumonia with autoimmune features (IPAF), screening for occult pSjD with MSGB and dry eye tests is recommended (85, 87, 89, 90).

**Figure 3:**
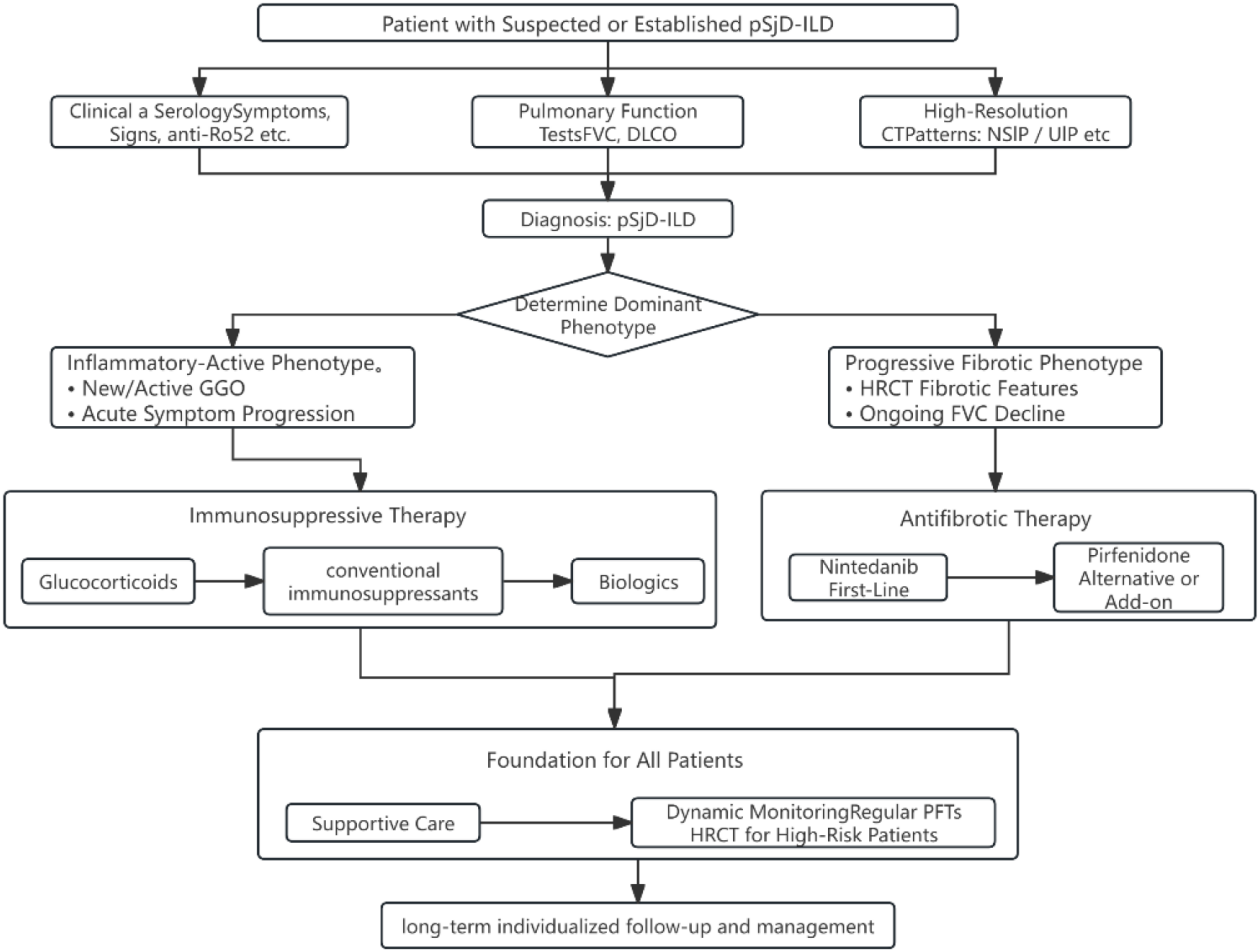
Phenotype-stratified treatment algorithm for primary Sjögren’s disease-associated interstitial lung disease (pSjD-ILD). This algorithm takes the dominant disease phenotype (inflammatory-active vs. progressive fibrotic) as the core decision-making basis. For patients with inflammatory-predominant disease, glucocorticoids (e.g., methylprednisolone pulse therapy followed by oral prednisone) combined with traditional immunosuppressants (e.g., cyclophosphamide, mycophenolate mofetil) are preferred for induction of remission. Biologic agents such as rituximab may be considered for refractory cases, especially those with serological or histological evidence of B-cell involvement. For patients with a progressive fibrotic phenotype, anti-fibrotic agents (e.g., nintedanib, pirfenidone) should be promptly added on the basis of basic immunosuppressive therapy, regardless of the HRCT pattern (UIP/NSIP). Additionally, all symptomatic patients require combined supportive care including pulmonary rehabilitation and oxygen therapy. Throughout the treatment process, dynamic adjustments to the therapeutic regimen should be made based on pulmonary function tests (FVC, DLCO) and HRCT results.

Diagnosis of pSjD-ILD lacks a unified global standard but relies on major guidelines: EULAR offers a concise rheumatology-based protocol for established pSjD patients (91), whereas ACR/CHEST provides a multidisciplinary comprehensive algorithm for initial evaluation of suspected CTD-ILD (84). We propose a phenotype-driven management algorithm (**Figure 3**) that emphasizes stratifying pSjD-ILD patients by dominant phenotype (inflammatory-active vs. progressive fibrotic) to guide all subsequent therapy.

## 5 Treatment strategies

Treatment of pSjD-ILD aims to control inflammation, inhibit fibrosis, preserve lung function, and improve quality of life. Management follows a phenotype-stratified approach (inflammatory-predominant vs. progressive fibrotic), with the critical caveat that no pSjD-ILD-specific RCTs exist; recommendations are extrapolated from other CTD-ILDs or small cohort studies (13, 92). **Table 2** summarizes treatment options and their evidence limitations.

**Table 2:** Overview of current treatment options for primary Sjögren’s disease-associated interstitial lung disease (pSjD-ILD).

Therapeutic category & agent	Key proposed efficacy in pSjD-ILD	Supporting evidence & common adverse events	Typical clinical context/Considerations
First-line Immunosuppression			
Glucocorticoids (e.g., Methylprednisolone pulse → oral Prednisone)	Rapid suppression of inflammatory activity; may improve symptoms and FVC in active, inflammatory-predominant disease.	Evidence: Guideline consensus, cohort data. AEs: Hyperglycemia, osteoporosis, infection risk.	Active, symptomatic disease (e.g., progressive dyspnea, new HRCT ground-glass opacities). Used for induction, with plans for timely steroid-sparing. Dosing: IV methylprednisolone 500–1000 mg/day for 3 d, followed by oral prednisone 0.5–1 mg/(kg·d) tapered to 5–10 mg/day over 4–8 weeks.
Conventional Immunosuppressants (csDMARDs) (e.g., Cyclophosphamide, Mycophenolate Mofetil)	Steroid-sparing agents; can stabilize lung function (FVC) and reduce acute exacerbation risk.	Evidence: Cohort studies (Cyclophosphamide, MMF). AEs: (CYC) Myelosuppression, hemorrhagic cystitis; (MMF) GI upset, potential teratogen.	Maintenance therapy after induction; or for progressive disease despite steroids. Choice depends on risk profile (e.g., CYC for severe/rapidly progressive cases). Dosing: CYC: IV 0.5–1 g/m² monthly for 6 cycles; MMF: 1–2 g/day orally in 2 divided doses.
Biologic & targeted therapies			
Rituximab (anti-CD20)	May improve symptoms and DLCO, particularly in patients with B-cell activation markers (e.g., anti-Ro52).	Evidence: Retrospective cohorts, expert consensus. AEs: Infusion reactions, increased infection risk; mandatory HBV/VZV screening before use.	Consider for active, refractory disease, especially with serological or histological evidence of B-cell involvement. Dosing: IV 1000 mg on days 1 and 15, repeated every 6 months for maintenance.
Antifibrotic Agents			
Nintedanib (Tyrosine kinase inhibitor)	Slows the rate of forced vital capacity (FVC) decline in patients with a progressive fibrosing phenotype, regardless of HRCT pattern (UIP/NSIP).	Evidence: Extrapolated from RCTs in other PF-ILDs (e.g., SSc-ILD). AEs: Diarrhea, nausea, liver enzyme elevation (dose-dependent).	Progressive fibrosing ILD phenotype (documented FVC decline over time). Often used on top of background immunosuppression. Dosing: 150 mg orally twice daily (dose reduction to 100 mg twice daily for adverse events).
Pirfenidone	May stabilize lung function in progressive fibrotic disease; data in pSjD-ILD are very limited.	Evidence: Small case series; extrapolated from IPF. AEs: Photosensitivity, GI upset, fatigue.	Alternative or add-on therapy for progressive fibrosis, particularly if nintedanib is not tolerated. Dosing: 500 mg orally three times daily, titrated to tolerance.
Other/Emerging biologics			
Abatacept (CTLA4-Ig)	May improve lung function in refractory cases; rationale based on targeting T-cell co-stimulation.	Evidence: Case reports/series. AEs: Increased infection risk, potential hypersensitivity.	Consider for active, refractory disease with inadequate response to B-cell-targeted therapy. Often used in combination. Dosing: Weight-based IV infusion at 0, 2, 4 weeks, then every 4 weeks (CTD-ILD reference regimen).
Tocilizumab (anti-IL-6R)	Potential to attenuate progression in patients with elevated systemic inflammation; strong signal in SSc-ILD.	Evidence: Anecdotal in pSjD; RCT evidence in SSc-ILD. AEs: Elevated lipids, GI perforation (rare), infection.	Rational off-label consideration in progressive disease with high inflammatory markers (e.g., CRP, ESR). Dosing: IV 8 mg/kg every 4 weeks, or 162 mg subcutaneously weekly.

AE, adverse event; CRP, C-reactive protein; CTD-ILD, connective tissue disease-associated interstitial lung disease; csDMARD, conventional synthetic disease-modifying antirheumatic drug; DLCO, diffusing capacity for carbon monoxide; FVC, forced vital capacity; GI, gastrointestinal; HBV, hepatitis B virus; HRCT, high-resolution computed tomography; ILD, interstitial lung disease; IPF, idiopathic pulmonary fibrosis; IV, intravenous; MMF, mycophenolate mofetil; NSIP, nonspecific interstitial pneumonia; PF-ILD, progressive fibrosing interstitial lung disease; RCT, randomized controlled trial; SSc-ILD, systemic sclerosis-associated ILD; UIP, usual interstitial pneumonia; VZV, varicella-zoster virus.

Overview of current therapeutic options for primary Sjögren’s disease-associated interstitial lung disease (pSjD-ILD), including therapeutic categories, key proposed efficacy, supporting evidence, common adverse events, and typical clinical application contexts. The table summarizes first-line immunosuppressants, biologic and targeted agents, antifibrotic drugs, providing a reference for phenotype-stratified and individualized treatment decisions.

### 5.1 Glucocorticoids and traditional immunosuppressants

For inflammatory-predominant pSjD-ILD (NSIP/OP with prominent GGOs), first-line treatment is immunosuppression with glucocorticoids plus csDMARDs. In acute or highly active disease, induction typically involves IV methylprednisolone pulse (500–1000 mg/day for 3 days) followed by oral prednisone (0.5–1 mg/kg/day) tapered to 5–10 mg/day over 4–8 weeks. Combination therapy with two pulse courses, oral prednisone, and tacrolimus (0.1 mg/kg/day) has been shown to improve FVC by a mean of 12% in CTD-ILD (93, 94).

csDMARDs function as steroid-sparing agents and long-term maintenance therapy, with selection guided by disease severity and comorbidities. Options include cyclophosphamide (IV 0.5–1 g/m² monthly for 6 cycles) for severe or rapidly progressive disease—though limited to DLCO improvement and associated with myelosuppression and hemorrhagic cystitis risks (94, 95); mycophenolate mofetil (1–2 g/day orally), offering comparable efficacy with a better safety profile and preferred for maintenance; and tacrolimus (0.1 mg/kg/day), which modulates T-cell activation, is effective in refractory cases often combined with biologics, and may improve sicca symptoms (94, 96).

Key limitation: Glucocorticoids plus csDMARDs have no proven efficacy in established fibrosis and are not recommended as monotherapy for progressive fibrotic pSjD-ILD (97, 98).

### 5.2 Biological disease-modifying antirheumatic drugs

bDMARDs target specific immune pathways and are indicated for refractory inflammatory-predominant pSjD-ILD after insufficient response to glucocorticoids and csDMARDs. All efficacy data are extrapolated from other CTD-ILDs or small pSjD-ILD cohorts, as no pSjD-ILD-specific RCTs exist.

Rituximab (anti-CD20) is the best-validated bDMARD for pSjD-ILD, particularly effective in anti-Ro52-positive patients and those with lymphocytic interstitial pneumonia (LIP) (52). Standard rheumatology dosing is 1000 mg IV on days 1 and 15, repeated every 6 months for maintenance (99). It reduces dyspnea VAS by 33 mm and increases DLCO by 7.6% in pSjD-ILD (97). Adverse events include infusion reactions and increased risk of serious respiratory infections; mandatory screening for hepatitis B and varicella-zoster is required before initiation. Key limitation: evidence is limited to retrospective pSjD-ILD cohorts; no RCTs available (52).

Other bDMARDs have limited or no pSjD-ILD data: Abatacept blocks T-cell co-stimulation and may improve lung function in refractory CTD-ILD, but its use in pSjD-ILD is restricted to case reports and small series (97); tocilizumab is effective in SSc-ILD (RCT data), yet experience in pSjD-ILD remains anecdotal and off-label (92); and anti-fractalkine targets monocyte/T-cell migration and showed a favorable safety profile in a phase I RA-ILD trial, but no pSjD-ILD data are available (98).

### 5.3 Anti-fibrotic therapy

Anti-fibrotic therapy is pivotal for progressive fibrotic pSjD-ILD, regardless of HRCT pattern, and should be initiated promptly in combination with background immunosuppression (glucocorticoids ± csDMARDs). All efficacy data are extrapolated from SSc-ILD RCTs, as no pSjD-ILD-specific trials exist; these agents target fibroblast activation and extracellular matrix deposition, the core drivers of end-stage respiratory failure (100, 101).

Nintedanib, a tyrosine kinase inhibitor, is the first-line anti-fibrotic for pSjD-ILD and the only agent approved for progressive fibrosing ILD of any cause, including CTD-ILD (102). It inhibits pro-fibrotic kinases (PDGFR, FGFR, VEGFR, Src), blocking fibroblast proliferation, migration, and collagen synthesis (102). The standard dosing is 150 mg orally twice daily (with dose reduction for adverse events). In SSc-ILD RCTs, nintedanib reduced annual FVC decline by 41.0 mL, with consistent efficacy across UIP and NSIP patterns; these benefits are extrapolated to pSjD-ILD with strong clinical consensus. Adverse events include dose-dependent gastrointestinal symptoms (diarrhea, nausea/vomiting) in approximately 60% of patients and mild liver enzyme elevation, though permanent discontinuation is rare (102).

Pirfenidone serves as an alternative anti-fibrotic for patients intolerant to nintedanib (e.g., severe gastrointestinal AEs). It inhibits TGF-β, TNF-α, and IL-1β, reducing fibroblast collagen synthesis and oxidative stress. Dosing is 500 mg orally three times daily, titrated to tolerance. Efficacy data from IPF and SSc-ILD phase II studies show stabilization of FVC in progressive fibrotic ILD, but experience in pSjD-ILD is limited to small case series (103). Adverse effects include photosensitivity, fatigue, and mild gastrointestinal upset, manageable with sun protection and dose adjustment (104).

Key limitation for all anti-fibrotics: No pSjD-ILD-specific RCTs exist, and long-term efficacy and safety in this population remain unknown (92).

### 5.4 Supportive and alternative management

Comprehensive supportive care is mandatory for all symptomatic pSjD-ILD patients, complementing immunosuppressive and anti-fibrotic therapy to reduce mortality and improve quality of life. Although all recommendations derive from CTD-ILD cohort data without pSjD-ILD-specific studies, several key interventions are well-established. Long-term oxygen therapy is indicated for resting (SpO_2_ <92%) or exercise-induced hypoxemia, alleviating dyspnea, improving exercise tolerance, and reducing hypoxic pulmonary hypertension risk. Pulmonary rehabilitation incorporates supervised breathing techniques, aerobic exercise, and muscle training, increasing six-minute walk distance by a mean of 50 meters and reducing dyspnea scores (1, 94). Nutritional support with a high-protein, high-calorie diet, including enteral supplementation for significant weight loss (>5% in six months), hypoalbuminemia, or severe dysphagia, addresses malnutrition as an independent mortality risk factor (105). Beyond pulmonary interventions, regular echocardiographic surveillance (annually or semi-annually) for high-risk patients enables early detection of pulmonary hypertension, with persistent elevation prompting targeted therapy (74). Infection prevention through influenza and pneumococcal vaccination is critical, as infections frequently trigger disease exacerbations (16). For patients with end-stage disease and refractory respiratory failure, lung transplantation remains a curative option; however, thorough pre-transplant multidisciplinary assessment of systemic autoimmune activity is essential to minimize post-transplant recurrence and graft rejection, acknowledging that CTD-ILD patients have nominally lower five-year post-transplant survival compared to those with idiopathic pulmonary fibrosis (106). Collectively, these supportive measures address the multifaceted burden of pSjD-ILD and should be integrated into routine management.

## 6 Prognostic evaluation and risk management

Prognosis in pSjD-ILD is associated with demographic, clinical, and serological factors. Key predictors of poor outcome include age ≥50 years, male sex, a UIP pattern, and low baseline lung function (FVC <70%, DLCO <60% predicted) (14, 68, 69, 107). Elevated serum KL-6, CEA and CA50 predict a worse pulmonary prognosis in pSS-ILD patients, with CEA and CA50 being independent predictors of fibrotic progression (77, 78). Complications including pulmonary hypertension, lymphoma, or malnutrition significantly increase mortality (1, 74, 105). Although no disease-specific prognostic score exists, integrated tools are used clinically. The GAP index estimates 3-year survival, while combining serum KL-6 with HRCT fibrosis scores improves accuracy (108).

Risk management focuses on proactive monitoring and early intervention. Guidelines recommend regular PFTs every 6–12 months for all pSjD patients (84). For those at high risk (e.g., anti-Ro52 positive or with declining DLCO), more frequent HRCT surveillance (e.g., every 6 months) is advised. A decrease in DLCO or the appearance of inspiratory crackles in seropositive patients should prompt consideration of early immunosuppressive therapy (15). Complication prevention requires routine echocardiography (assessing TAPSE and sPAP) for early detection of pulmonary hypertension and appropriate vaccination against influenza and pneumococcal disease to reduce infection risk (74, 94).

## 7 Current limitations in pSjD-ILD research and clinical practice

Despite advances in understanding pSjD-ILD, several critical limitations persist that hinder optimal clinical care and research progress (16, 22, 92):

### 7.1 Lack of disease-specific clinical evidence

All therapeutic recommendations are extrapolated from other CTD-ILDs or small cohort studies; no phase II/III RCTs have evaluated immunosuppressive or anti-fibrotic agents specifically in pSjD-ILD, precluding definitive conclusions on optimal dosing, efficacy, and long-term safety (92).

### 7.2 Absence of standardized diagnostic and prognostic tools

No unified global diagnostic standard exists for pSjD-ILD, and no disease-specific prognostic score has been validated. Clinicians therefore rely on extrapolated tools such as the GAP index, leading to heterogeneous risk stratification and monitoring practices (16).

### 7.3 Incomplete biomarker validation and heterogeneous study designs

Although several biomarkers (e.g., KL-6, Angptl2, CA 50) show promise, validated cutoffs are lacking for most monitoring markers (e.g., CA 19-9, CA 125), and no biomarker panel has been prospectively validated for treatment response assessment. Meanwhile, epidemiological and clinical research is constrained by small sample sizes, variable diagnostic criteria, and inconsistent follow-up, limiting generalizability and complicating meta-analysis (76, 78).

### 7.4 Unclear subtype natural history and limited evidence for combination therapy

The trajectory of individual pSjD-ILD subtypes (e.g., LIP, fibrotic NSIP, UIP) remains undefined, with no data on factors driving progression or subtype conversion; data are also lacking on optimal combination regimens (e.g., rituximab plus nintedanib) in progressive fibrotic pSjD-ILD, and the role of bDMARDs alongside anti-fibrotics is unestablished (92).

Collectively, these gaps underscore the urgent need for pSjD-ILD-specific research initiatives, standardized data collection, and international collaboration to advance evidence-based care.

## 8 Research progress and future prospects

Recent translational research has uncovered novel pathogenic mechanisms and therapeutic targets in pSjD-ILD, paving the way for precision medicine approaches (47, 53). Single-cell sequencing has identified a pathogenic pro-inflammatory fibroblast subset (CXCL10^+^CCL19^+^) in pSjD-ILD lungs, with its prevalence correlating with fibrosis severity, offering a potential cell-specific therapeutic target (54). Epigenetic studies have revealed MBD2-mediated methylation of the AHR promoter as a key driver of fibrosis; this novel target is now being explored for small-molecule inhibition (53). The luciferase immunoprecipitation system (LIPS) enables quantitative monitoring of anti-Ro52 and other autoantibodies, providing a dynamic biomarker for treatment response and FVC improvement (47).

Building on these advances, key research priorities have emerged to address current limitations. First, pSjD-ILD-specific phase II/III RCTs are urgently needed to validate the efficacy and safety of both immunosuppressive agents (e.g., rituximab) and anti-fibrotics (e.g., nintedanib) in this population. Second, the development and validation of a pSjD-ILD-specific prognostic score integrating demographic, clinical, serological, and radiological factors would enable more accurate risk stratification. Third, biomarker panels with standardized cutoffs must be validated for diagnosis, risk stratification, and treatment response monitoring. Fourth, prospective studies are required to define the natural history of pSjD-ILD subtypes (e.g., LIP, fibrotic NSIP, UIP), facilitating subtype-specific treatment strategies. Fifth, emerging therapeutic targets including MBD2, the AHR-PTEN axis, and gut-lung axis mediators warrant further exploration for precision medicine applications. Finally, establishing international multicenter registries is essential to standardize data collection, address study heterogeneity, and facilitate collaborative research.

Realizing these priorities will require coordinated efforts through standardized multidisciplinary team care and robust international collaboration, ultimately translating mechanistic insights into improved clinical outcomes for pSjD-ILD patients (87, 90).

## 9 Conclusion

Primary Sjögren’s disease-associated interstitial lung disease (pSjD-ILD) affects 13–20% of pSjD patients, with anti-Ro52 seropositivity, advanced age, and male sex as the most robust poor prognostic factors. Phenotype stratification into inflammatory-predominant and progressive fibrotic subtypes is the cornerstone of pSjD-ILD clinical management, guiding individualized immunosuppressive and anti-fibrotic therapy—though all current therapeutic recommendations rely on extrapolation from other CTD-ILDs due to the lack of pSjD-ILD-specific RCTs.

Clinically, multidisciplinary team care, regular dynamic monitoring (combining PFTs, echocardiography and stratified biomarkers), and early intervention for complications (e.g., pulmonary hypertension) are critical to improving patient outcomes.

The field of pSjD-ILD currently faces key unmet needs, including the lack of disease-specific clinical trials, standardized diagnostic/prognostic tools, and validated biomarker panels. Future research should prioritize these gaps, alongside exploration of novel therapeutic targets (e.g., MBD2, AHR-PTEN axis) and international multicenter collaboration, to translate mechanistic insights into precision medicine and improve long-term survival for pSjD-ILD patients.
